# From algorithms to operating room: can large language models master China’s attending anesthesiology exam? A cross-sectional evaluation

**DOI:** 10.1097/JS9.0000000000003406

**Published:** 2025-09-04

**Authors:** Qiyu He, Zhimin Tan, Wang Niu, Dongxu Chen, Xian Zhang, Feng Qin, Jiuhong Yuan

**Affiliations:** a Department of Urology and Andrology Laboratory, West China Hospital, Sichuan University, Chengdu, Sichuan Province, China; b Department of Anesthesiology, West China Hospital, Sichuan University, Chengdu, Sichuan Province, China; c Department of Anesthesiology, West China Second Hospital, Sichuan University, Chengdu, Sichuan Province, China; d Key Laboratory of Birth Defects and Related Diseases of Women and Children (Sichuan University), Ministry of Education, Chengdu, Sichuan Province, China

**Keywords:** anesthesiology, attending physician examination, ChatGPT, DeepSeek, large language models

## Abstract

**Objective:**

The performance of large language models (LLMs) in complex clinical reasoning tasks is not well established. This study compares ChatGPT (GPT-3.5 and GPT-4) and DeepSeek (DeepSeek-V3 and DeepSeek-R1) in the Chinese anesthesiology attending physician examination (CAAPE), aiming to set artificial intelligence (AI) benchmarks in medical assessments and enhance AI-driven medical education.

**Methods:**

This cross-sectional study assessed 4 iterations of 2 major LLMs on the 2025 CAAPE question bank (5647 questions). Testing employed diverse querying strategies and languages, with subgroup analyses by subspecialty, knowledge type, and question format. The focus was on LLM performance in clinical and logical reasoning tasks, measuring accuracy, error types, and response times.

**Results:**

DeepSeek-R1 (70.6–73.4%) and GPT-4 (68.6–70.3%) outperformed DeepSeek-V3 (53.1–55.5%) and GPT-3.5 (52.2–55.7%) across all strategies. System role (SR) improved performance, while joint response degraded it. DeepSeek-R1 outperformed GPT-4 in complex subspecialties, reaching peak accuracy (73.4%) under SR combined initial response. Generative Pre-trained Transformers (GPT) models performed better with English than Chinese queries. All models excelled in basic knowledge and Type A1 questions but struggled with clinical scenarios and advanced reasoning. Despite DeepSeek-R1’s stronger performance, its response time was longer. Errors were primarily logical and informational (over 70%), with more than half being high-risk clinical errors.

**Conclusion:**

LLMs show promise in complex clinical reasoning but risk critical errors in high-risk settings. While useful for education and decision support, their error potential must be carefully assessed in high-stakes environments.

**HIGHLIGHTS**

- **LLM performance in anesthesiology exams**: DeepSeek-R1 excels in accuracy and reasoning but is slower, while GPT-4 balances speed and performance.
- **Complex reasoning challenges**: All models falter in clinical scenario-based and high-order reasoning tasks, with DeepSeek-R1 and GPT-4 performing better but prone to logical errors, limiting perioperative decision-making simulation.
- **Language-specific barriers**: GPT models outperform in English over Chinese, underscoring the need for localized LLM optimization in non-English medical contexts.
- **Prompting strategy effects**: SR prompting enhances accuracy, whereas repeated questioning reduces it, suggesting an overthinking effect in LLMs.
- **Clinical and research implications**: Advanced LLMs show potential in anesthesiology education and decision support, but deficiencies in complex reasoning and language-specific performance necessitate targeted optimization for reliable use in diverse, high-stakes medical settings.

## Introduction

In recent years, large language models (LLMs) based on Generative Pre-trained Transformers (GPT) have noted a widespread use, particularly in healthcare and education. Through pretraining on extensive corpora and deep learning techniques, LLMs exhibit substantial potential in natural language understanding, text generation, and task reasoning^[1]^.

Since the release of GPT-4, LLMs have been widely applied to medical licensing exams (MLEs) and other academic tasks. For example, GPT-4 achieved 81.1% accuracy in the US MLE^[2]^, 72.7% in the Chinese MLE^[3]^, and 89.2% in the Japanese MLE^[4]^, all exceeding the entrance thresholds. These outcomes support further artificial intelligence (AI) integration in healthcare and medical education. Despite increasing interest, AI models still face linguistic and cultural challenges in Chinese MLEs^[5,6]^, primarily due to limitations in the current LLMs’ capacity for accurate medical terminology recognition, semantic parsing, and contextual adaptation^[7,8]^. These competencies are essential for handling the specialized language required in such exams.

Crucially, evidence remains limited that LLMs can reliably support complex clinical scenarios requiring high-level reasoning. In particular, current models struggle with constructing coherent reasoning chains, identifying implicit or uncertain information, and interpreting causal relationships within clinical contexts^[9]^. These limitations hinder their ability to replicate the systematic judgment essential for real-world medical decision-making. This gap hinders LLMs’ potential in clinical decision support. Therefore, to what extent can AI assist in exams requiring advanced clinical judgment? Unlike entrance exams, attending physician exams (APEs) assess medical knowledge and clinical reasoning, with a focus on decision-making skills^[10,11]^. In anesthesiology, a high-risk specialty, the APE especially evaluates competencies such as clinical reasoning, decision-making, and crisis management, particularly in perioperative care^[12–15]^. As such, assessing LLMs’ performance in anesthesiology exams is vital for optimizing AI applications in medical education and clinical decision support.

This study assesses the performance of LLMs, including ChatGPT and DeepSeek, on the Chinese Anesthesiologist Associate Physician Examination (CAAPE). It specifically examines their bilingual medical knowledge and clinical reasoning capabilities. A novel evaluation framework is introduced, incorporating multilingual input, repeated querying, role-based prompting, scenario classification, reasoning analysis, and error-risk stratification. This approach aims to address a critical gap in existing research, offering insights into the potential of AI in medical education and clinical decision-making, with broader implications for the future of intelligent healthcare systems.

## Materials and methods

### Source and classification of questions

The CAAPE is a national healthcare professional qualification exam, developed by the Health Human Resources Development Center, National Health Commission, P.R. China (NHCC). CAAPE exam content is copyrighted by NHCC, and due to national policies, questions cannot be collected or published (https://www.21wecan.com/wsrcw/c100193/202404/1001730.shtml). To accurately simulate the CAAPE, this study uses the official question bank designated by NHCC. The question bank was created by the National Health Professional Qualification Exam Textbook Expert Committee, adhering strictly to the latest exam syllabus and aligning with the actual distribution of exam subjects and question types (https://www.21wecan.com/wsrcw/c100198/202411/1002133.shtml).

**Figure 1.**
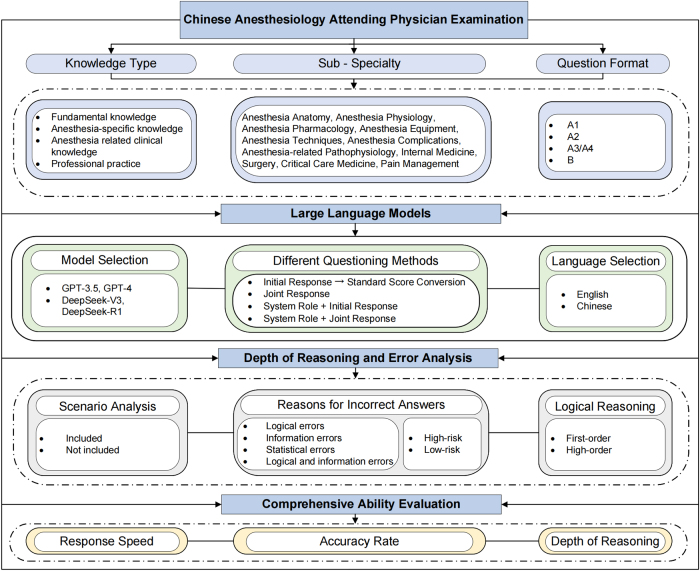
Technical route of this study.

The CAAPE is a pass or fail exam with 4 units, totaling 400 points. The first three units consist of 100 single-choice questions (1 point each), while the fourth unit includes both single- and multiple-choice questions (2 points each). Incorrect or incomplete answers lead to point deductions, with a minimum score of zero. A total score above 240 points, with at least 60 points per unit, is required to pass. To evaluate LLM performance, all questions were independently categorized by two experienced anesthesiologists according to CAAPE standards. Disagreements were resolved through discussion among all authors. Ultimately, the questions were categorized into 11 subspecialties (Anesthesia Anatomy, Anesthesia Physiology, Anesthesia Pharmacology, Anesthesia Equipment, Anesthesia Techniques, Anesthesia Complications, Anesthesia-Related Pathophysiology, Internal Medicine, Surgery, Critical Care, and Pain Medicine), 4 knowledge types (Unit 1: Basic Knowledge, Unit 2: Anesthesia Specialty Knowledge, Unit 3: Anesthesia-Related Clinical Knowledge, and Unit 4: Professional Practice Knowledge), and 4 question formats (A1: Single Best Answer, A2: Case Summary Best Answer, A3/A4: Case Set Best Answer, and B: Standard Combination Questions). This cross-sectional study adheres to the STROCSS criteria, ensuring rigorous and standardized reporting^[16]^. The study methodology is illustrated in Figure 1.

To avoid overlap with LLM training data (cutoff dates: GPT-3.5: Sept 2021; GPT-4: Jun 2024; DeepSeek-V3: Dec 2023; and DeepSeek-R1: Jul 2024), we used the 2025 CAAPE question bank, publicly released in December 2024. It contains 5647 text-based questions, excluding images and tables. Sub-specialties and knowledge points were extracted from the CAAPE syllabus (https://www.21wecan.com/wsrcw/KSDAXYL/list.shtml) and matched with 5647 mock questions from the official question bank (Supplemental Digital Content Table S1, Available at, http://links.lww.com/JS9/F6), showing high consistency with the syllabus requirements. As the data are publicly accessible, institutional review board approval was not required.

### LLM selection and questioning strategy

This study tested two representative LLMs and their iterations: the ChatGPT series (GPT-3.5 and GPT-4) and the DeepSeek series (DeepSeek V3 and DeepSeek-R1), with all tests completed by February 2025. Two authors oversaw the problem-solving tasks, with all authors reviewing the results.

To ensure accuracy, questions were posed in the language used by the LLM’s training data. The CAAPE questions are in Chinese. For the DeepSeek series, trained on Chinese data, we used the original Chinese questions. For the ChatGPT series, trained on English data, questions were posed separately in Chinese and English. To ensure translation accuracy, two experienced anesthesiologists independently translated the questions into English using ChatGPT, followed by a back-translation process. The English version was then translated back into Chinese and compared with the original questions. If discrepancies were found, the English version was revised and re-validated through back-translation until the final Chinese text matched the original. In cases of disagreement between the translators, they collaboratively resolved the issue. To minimize prior-dialogue interference, new windows were used for English questions. Related questions were asked consecutively in the same session, while unrelated ones were assigned separate sessions to preserve query independence. The overall accuracy, bilingual correctness, monolingual correctness, and logical consistency (where both languages were either correct or incorrect) of GPT-3.5 and GPT-4 were compared across different languages. Error patterns in various language contexts were analyzed, and subgroup analysis based on four units was conducted.

After obtaining the initial response (IR) from the LLM, it was compared to the standard answer to assess accuracy. Previous studies indicate that repeated questioning may lead to inconsistencies due to overthinking^[17,18]^, intermediate forgetting^[19]^, and cognitive uncertainty^[20]^. Therefore, a repeated questioning strategy was employed: if the second response matches the IR, the process ends, and the joint response (JR) is compared to the correct answer; if the responses differ, the question is asked a third time. If two of three responses align, these are used as the JR and evaluated against the standard answer. If all three responses differ, the JR is marked incorrect. The IR primarily assesses the LLM’s potential to pass the CAAPE in an anesthesiologist exam scenario, while the JR reflects overall accuracy (the proportion of questions with at least two correct answers), better representing the LLMs’ potential in medical education^[3,21]^. To assess passing potential, accuracy under IR conditions was converted into standardized scores, estimating the LLMs’ performance in an anesthesiologist exam context.

**Figure 2.**
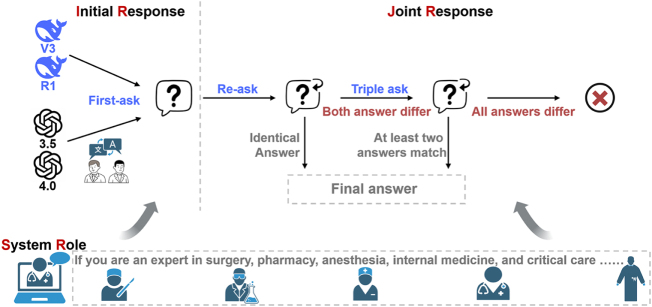
Schematic of LLMs questioning strategies. LLM, large language models.

Role-playing prompts have been recognized as a key strategy for enhancing chatbot accuracy^[22,23]^. To assess LLM response quality under different conditions, we introduced a dynamic system role (SR) prompt based on subspecialty classification for each question (e.g., “You are an expert in anesthesia pathophysiology, please select the best option and explain why”). Response times for each LLM were recorded, with the JR time calculated as the average of repeated responses. The question strategy diagram is shown in Figure 2. The temperature parameter, which influences the randomness of the responses, was set at the default value of 0.7 for both series to maintain balanced response variability^[24,25]^. The accuracy data of the four LLMs under different questioning strategies (IR, JR, SR + IR, and SR + JR) were extracted to calculate the overall mean and standard deviation. Effect sizes (Cohen’s *d*) were then calculated using the following formula to assess the practical significance of the accuracy differences between the models.

$$SD_{Pooled}=\sqrt{\frac{\left(n_{1}-1\right)\cdot {SD}_{1}^{2}+\left(n_{2}-1\right)\cdot {SD}_{2}^{2}}{n_{1}+n_{2}-2}}d=\frac{M_{1}-M_{2}}{SD_{Pooled}}$$

### Scenario analysis and logical reasoning

To assess LLMs’ scenario analysis and logical reasoning in clinical decision support, two experienced anesthesiologists classified questions by clinical context and cognitive complexity, discrepancies were resolved through author team discussions, and κ values were calculated for consistency^[26]^. Questions involving patient-specific information (e.g., demographics, medical history, surgery, and anesthesia details) necessary for answering were categorized as “Clinical Scenario”^[27]^. Questions based on theoretical knowledge or patient information not essential for answering were categorized as “Non-Clinical Scenario” (Supplemental Digital Content Table S2, Available at, http://links.lww.com/JS9/F6). Following prior study designs, questions were classified as first-order or high-order based on logical reasoning^[27,28]^. First-order questions involve simple recall, answer selection, or truth determination, while high-order questions require more complex reasoning, such as diagnostic estimation or guideline application (Supplemental Digital Content Table S2, Available at, http://links.lww.com/JS9/F6).

### Visualization of LLM performance comparison

GPT-3.5, GPT-4, DeepSeek-V3, and DeepSeek-R1 were ranked based on overall accuracy, performance on clinical scenario analysis and high-order reasoning, and response speed, with scores of 4, 3, 2, and 1, respectively. A radar chart was used to visually compare their performance in the CAAPE across accuracy, logical reasoning, and speed.

### Error analysis

During the LLM problem-solving tasks, two authors independently identified the causes of incorrect answers, categorizing them as logical, informational, statistical errors, or ambiguity. Discrepancies were resolved through discussion among all authors. The author team further classified LLM errors based on clinical severity. Errors meeting any of the following criteria were categorized as high clinical risk: (1) diagnostic or anesthetic assessment errors; (2) treatment or procedural errors; (3) critical dosing or calculation errors; (4) key information errors; (5) emergency management errors; and (6) errors involving high-risk populations (e.g., children, pregnant women). Errors not directly affecting patient safety or treatment outcomes were classified as low clinical risk.

### Sample size calculation

This study referenced existing data on model accuracy (GPT-3.5: 54–65%^[3,29]^, GPT-4: 63–77%^[30,31]^, DeepSeek-V3: 60–64%^[32,33]^, DeepSeek-R1: 60–82%^[29,34]^, with inter-model differences of 2–30%^[27,34]^). Based on this, GPT-3.5 accuracy was set at 60%, GPT-4 at 69%, DeepSeek-V3 at 62%, and DeepSeek-R1 at 71%. The minimum clinically significant difference (group-wise difference) was 5%. The effect size (*ω*) was calculated using the formula below, with $p_{i}$ representing the expected accuracy for each group and $\overset{ˉ}{p}$ the overall mean accuracy, yielding an effect size of *ω* = 0.114.

$$\omega =\sqrt{\overset{k}{\underset{i=1}{\sum }}\frac{{\left(p_{i}-\overset{ˉ}{p}\right)}^{2}}{\overset{ˉ}{p}}}$$

The LLM responses in this study were binary (*R* = 2) and evaluated using four models (*C* = 4). Degrees of Freedom (df) were calculated as 3:

$$df=\left(R-1\right)\times \left(C-1\right)$$

Using GPower 3.1.9.7, a *χ*^2^ test (Goodness-of-fit: Contingency tables) was set with *α* = 0.05 and 1−*β* = 0.95. With an effect size of *ω* = 0.114 and df = 3, the total sample size was calculated as 1322, with an average of 331 per model and an actual power of 0.95 (Supplemental Digital Content Figure S1, Available at, http://links.lww.com/JS9/F6). To ensure statistical power, 5647 questions were included, exceeding the minimum sample size estimate.

### Statistical methods

Chi-square (*χ*^2^) tests were employed to assess accuracy differences between GPT-3.5, GPT-4, DeepSeek-V3, and DeepSeek-R1 across four testing strategies. Subgroup analyses were conducted based on knowledge types and question formats. *χ*^2^ tests also compared accuracy in clinical vs. non-clinical scenarios, and first-order vs. high-order questions, and low-risk vs. high-risk errors, with Bonferroni correction applied to control for multiple comparisons. Accuracy differences between GPT-3.5 and GPT-4 in both Chinese and English were analyzed, alongside logical consistency. Error analysis was performed using descriptive statistics to categorize and calculate error types and proportions. Thinking time differences were evaluated via one-way ANOVA. κ statistics were used to measure categorization consistency, with statistical significance set at *P* < 0.05.

## Results

### Overview of questions

**Figure 3.**
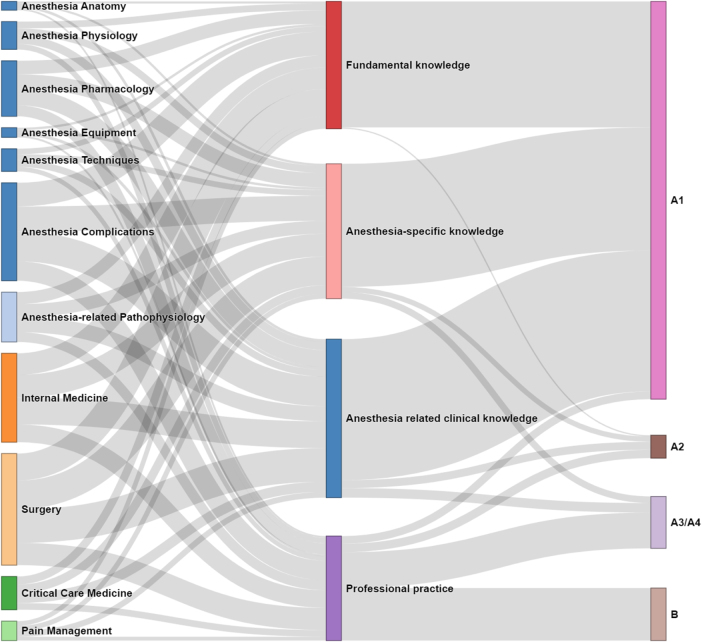
The Sankey diagram of question classifications: 11 sub-specialties, 4 knowledge types, and 4 question formats.

This study analyzed the complete set of 5647 choice questions from the 2025 CAAPE simulation question bank, with the distribution shown in the Sankey diagram (Fig. 3). Anesthesia complications (1053) and Surgery (1200) comprised the majority of subspecialty questions. Anesthesia-related clinical knowledge accounted for the largest knowledge category, with 1704 questions, underscoring anesthesiology’s critical role in procedures and patient management. Although professional practice also pertains to clinical application, it emphasizes the technical and procedural aspects of anesthesia. The most common question format was the A1 single-best answer type, with 4278 questions. Categorization consistency was high, with a κ value of 0.935 (*P* < 0.001).

### LLM’s performance under different questioning strategies and subgroups

After standard score conversion, only DeepSeek-R1 (285.0) and GPT-4 (278.3) passed the CAAPE. U1 (Fundamental Knowledge) had the highest scores across all models, while U4 (Professional Practice) had the lowest (Supplemental Digital Content Figure S1, Available at, http://links.lww.com/JS9/F6). DeepSeek-R1 and GPT-4 outperformed DeepSeek-V3 and GPT-3.5 across all questioning strategies (IR, JR, SR + IR, and SR + JR) in accuracy (Table 1). The effect sizes from pairwise comparisons of the overall accuracy of the four LLMs show that, except for the comparison between GPT-3.5 and DeepSeek-V3, all other Cohen’s *d* values have absolute values greater than 0.8, indicating that the accuracy differences between the models are of practical significance (Supplemental Digital Content Table S3, Available at, http://links.lww.com/JS9/F6)^[35]^. SR consistently improved model performance, with DeepSeek-R1’s accuracy rising from 71.2% (IR) to 73.4% (SR + IR). However, the JR strategy did not enhance accuracy and led to a performance decline across all models, even with SR. LLMs performed better on basic knowledge and A1-type questions compared to other categories (Supplemental Digital Content Table S4, Available at, http://links.lww.com/JS9/F6). For instance, DeepSeek-R1’s accuracy on basic knowledge under SR + IR reached 78.7% (*N* = 1077), an 8.1 percentage point increase over professional practice accuracy (70.6%) (*P* < 0.001). Similarly, for A1-type questions, its accuracy was 74.7% (*N* = 3193) under SR + IR, 7.8 percentage points higher than for B-type questions (66.9%) (*P* = 0.008). Across models, SR + IR yielded the highest accuracy, while JR produced the lowest (Supplemental Digital Content Table S4, Available at, http://links.lww.com/JS9/F6).

**Table 1:** Accuracy of ChatGPT and DeepSeek under different questioning strategies

Accuracy	GPT-3.5, *n* (%)	GPT-4, *n* (%)	DeepSeek-V3, *n* (%)	DeepSeek-R1, *n* (%)	*P* value
IR	3078 (54.5)	3925 (69.5)	3010 (53.3)	4021 (71.2)	<0.001
JR	2948 (52.2)	3874 (68.6)	2999 (53.1)	3987 (70.6)	<0.001
SR + IR	3145 (55.7)	3970 (70.3)	3134 (55.5)	4145 (73.4)	<0.001
SR + JR	3066 (54.3)	3959 (70.1)	3100 (54.9)	4105 (72.7)	<0.001

IR, initial response; JR, joint response (the question is asked three times, and if two or more responses are identical, the answer is considered correct); SR, system role. *P* values were derived from *χ*^2^ tests comparing accuracy rates across the four models within each testing condition, and *n* represents the number of correct answers.

**Figure 4.**
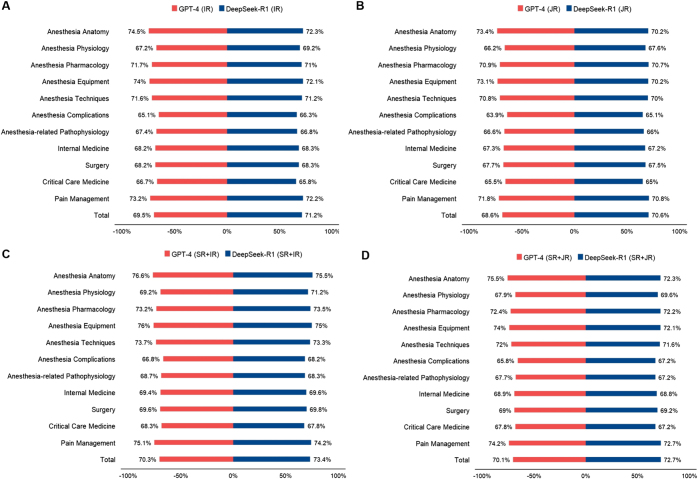
Accuracy for GPT-4.0 and DeepSeek-R1 classified by 11 sub-specialties. (a) IR, (b) JR, (c) IR with SR assignation, and (d) JR with SR assignation. IR, initial response; JR, joint response, SR, system role.

In anesthesia-related knowledge, DeepSeek-V3 scored 54.0% (SR + IR) and 52.0% (JR), slightly outperforming GPT-3.5 (53.9% and 50.5%). DeepSeek-R1 outperformed GPT-4 across 11 medical subspecialties, especially in complex clinical areas (Fig. 4). Under the SR + IR, DeepSeek-R1 achieved 73.4% accuracy, 3.1 percentage points higher than GPT-4. In high-difficulty fields like anesthesia physiology, DeepSeek-R1’s advantage was more pronounced, scoring 71.2%. DeepSeek-R1 demonstrated greater consistency under SR + IR, particularly in intensive care.

Response times varied significantly across LLMs and questioning strategies (Table 2). GPT-3.5 was the fastest (1.2 ± 0.3 to 2.3 ± 1.1s), while DeepSeek-R1 had the longest times (42.9 ± 2.6 to 49.7 ± 8.5 s). The shortest times were seen under the IR strategy, with SR and JR, particularly JR, increasing processing time. For example, DeepSeek-R1’s response time under SR + JR (49.7 ± 8.5 s) was significantly longer than other strategies (*P* < 0.001).

**Table 2:** Duration of contemplation for the question

Category	IR	JR*	SR + IR	SR + JR*	*P* value
GPT-3.5, s (mean ± SD)	1.2 ± 0.3	1.9 ± 0.5	1.5 ± 0.4	2.3 ± 1.1	<0.001
GPT-4, s (mean ± SD)	5.4 ± 0.8	6. 6 ± 2.3	6.1 ± 1.5	7.0 ± 2.9	<0.001
DeepSeek-V3, s (mean ± SD)	10.6 ± 1.7	12.1 ± 3.4	11.4 ± 2.1	12.8 ± 4.2	<0.001
DeepSeek-R1, s (mean ± SD)	42.9 ± 2.6	47.8 ± 6.1	44.9 ± 5.0	49.7 ± 8.5	<0.001

IR, initial response; JR, joint response (the question is asked three times, and if two or more responses are identical, the answer is considered correct); SR, system role.

* Select the average value of the time for each response. *P* value was evaluated using one-way ANOVA.

### Clinical scenario analysis and logical reasoning performance

A total of 1853 clinical scenario analysis questions and 2538 high-order logical reasoning questions were identified (Table 3). The κ value for the two anesthesiologists’ classification of Clinical scenario analysis and logical reasoning was 0.95, with accuracy exceeding 98% when compared to the final classification (Supplemental Digital Content Tables S5–S6, available at, http://links.lww.com/JS9/F6). Overall, accuracy on non-clinical scenario analysis (*N* = 3794) exceeded that on clinical scenarios (*N* = 1853), and first-order logical reasoning (*N* = 3109) outperformed high-order reasoning (*N* = 2538). Specifically, DeepSeek-R1 and GPT-4 outperformed DeepSeek-V3 and GPT-3.5 in clinical analysis and high-order reasoning, with DeepSeek-R1 slightly ahead of GPT-4. Conversely, GPT-3.5 excelled over DeepSeek-V3 on non-clinical scenario analysis (57.2% vs. 54.7%) and first-order reasoning (56.1% vs. 53.5%). After Bonferroni correction, all model differences were statistically significant (*P* < 0.001).

**Table 3:** Performance of scenario analysis and logical reasoning

Category of questions	GPT-3.5^a^, *n* (%)	*P* value^b^	GPT-4^a^, *n* (%)	*P* value^b^	DeepSeek- V3^a^, *n* (%)	*P* value^b^	DeepSeek- R1^a^, *n* (%)	*P* value^b^	*P* value^c^
Scenario analysis									
Included	907	<.001	1257	.047	934	.003	1318	.862	<0.001
(*N* = 1853)	(48.9)		(67.8)		(50.4)				
							(71.1)		
Not included	2171		2668		2076		2703		<0.001
(*N* = 3794)	(57.2)		(70.3)		(54.7)				
							(71.2)		
Logical reasoning									
First-order	1743	.307	2195	.018	1662	.007	2223	.228	<0.001
			(70.6)						
					(53.5)		(71.5)		
(*N* = 3109)	(56.1)								
High-order	1335		1730		1348		1798		<0.001
			(68.2)						
					(53.1)		(70.8)		
(*N* = 2538)	(52.6)								

^a^ Performance metrics are presented as the number of correct answers (Percentage of total questions in each category).

^b^ *P* values were calculated using *χ*^2^ tests (Longitudinal comparison within the same LLM between Clinical Scenario and Non-Clinical Scenario, or between First-order and High-order Logical Reasoning).

^c^ *P* values were calculated using Bonferroni correction for multiple comparisons to control the error rate (a lateral comparison of different LLMs under the same Clinical Scenario or Logical Reasoning conditions).

### Performance differences between English and Chinese

The performance of GPT-3.5 and GPT-4 across four knowledge domains was compared in English and Chinese question formats (Supplemental Digital Content Table S7, Available at, http://links.lww.com/JS9/F6). In English, GPT-3.5 and GPT-4 achieved accuracies of 54.5% and 69.5%, respectively, while in Chinese, their accuracies dropped to 53.2% and 68.4%. In the English questioning environment, GPT-4 outperformed GPT-3.5 across all anesthesiology knowledge areas, with accuracy rates of 69% and 53%, respectively. GPT-4’s accuracy in basic knowledge was 75%, higher than GPT-3.5’s 58%, while both models had stable accuracy of around 65% in professional practice, with GPT-4 surpassing GPT-3.5’s 51%. These results suggest GPT-4 demonstrates better understanding and application of core knowledge in English. A further analysis of logical consistency between Chinese and English questions showed self-consistency rates of 77.3% for GPT-3.5 and 77.1% for GPT-4. Specifically, 12.0% of GPT-3.5’s responses were correct only in English, and 10.7% only in Chinese; for GPT-4, the rates were 12.0% and 10.9%.

### Error analysis

Among the LLMs in the ChatGPT and DeepSeek series, DeepSeek-V3 had the lowest accuracy. However, GPT-3.5’s overall error rate in Chinese reached 46.8%, surpassing that of DeepSeek-V3 (Table 4). Errors across all models were primarily due to logical and informational inaccuracies, with statistical errors being minimal (less than 2%). Notably, while DeepSeek-R1 had the highest proportion of informational errors, the total number (650) was still lower than that of any other model. Error analysis found no inaccuracies caused by the translation process from Chinese to English. On the contrary, GPT models performed better with English prompts than with Chinese. Error classification based on clinical severity revealed that more than half of the errors in all LLMs were high clinical risk errors, directly impacting patient safety or treatment outcomes (Supplemental Digital Content Table S8, Available at, http://links.lww.com/JS9/F6).

**Table 4:** Reasons for incorrect answers

Category	GPT-3.5 (Chinese)	GPT-3.5 (English)	GPT-4 (Chinese)	GPT-4 (English)	DeepSeek-V3	DeepSeek-R1
Logical errors, *n* (%)	1192 (21.1)	1159 (20.5)	728 (12.9)	707 (12.5)	1077 (19.1)	569 (10.1)
Information errors, *n* (%)	948 (16.8)	875 (15.5)	643 (11.4)	583 (10.3)	1055 (18.7)	650 (11.5)
Statistical errors, *n* (%)	106 (1.9)	98 (1.7)	71 (1.3)	65 (1.2)	105 (1.9)	65 (1.2)
Ambiguous answer, *n* (%)	396 (7.0)	437 (7.7)	340 (6.0)	367 (6.5)	400 (7.1)	342 (6.1)
Total, *n* (%)	2642 (46.8)	2569 (45.5)	1782 (31.6)	1722 (30.5)	2637 (46.7)	1626 (28.8)

### Overall performance comparison

**Figure 5.**
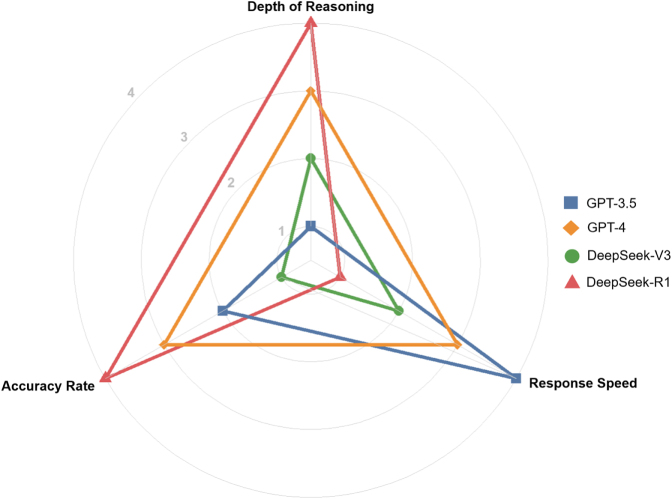
Comparative performance of AI models in accuracy, response speed, and reasoning depth. AI: artificial intelligence.

The radar chart (Fig. 5) highlights the differences in performance across the four LLMs in terms of accuracy, logical reasoning, and thinking speed. In accuracy (based on IR correctness), DeepSeek-R1 achieved the highest score (4 points, 71.2%), significantly outperforming other models. GPT-4 scored 3 points (69.5%), while GPT-3.5 (55.7%) and DeepSeek-V3 (55.5%) scored 2 and 1 points, respectively. In logical reasoning (based on high-order reasoning accuracy), DeepSeek-R1 led with 4 points (70.8%), followed by GPT-4 with 3 points (68.2%). DeepSeek-V3 (54.5%) and GPT-3.5 (53.5%) scored 2 and 1 points, respectively. For thinking speed, GPT-3.5 was the fastest (4 points, 1.2 s), GPT-4 had moderate speed (3 points, 5.4 s), while DeepSeek (10.6 s) and DeepSeek-R1 (42.9 s) were slower, earning 2 and 1 points, respectively.

## Discussion

### Key findings

This study evaluated the performance of GPT-3.5, GPT-4, DeepSeek-V3, and DeepSeek-R1 in the CAAPE. After standard score conversion, only DeepSeek-R1 and GPT-4 passed the 2025 CAAPE, with most incorrect answers classified as high clinical risk. DeepSeek-R1 performed better in accuracy and reasoning but was slower, while GPT-4 showed balanced performance with overall superior results. DeepSeek-V3 lagged in both accuracy and speed, and GPT-3.5, though faster, performed weaker in accuracy and reasoning. The SR strategy boosted performance by activating relevant knowledge networks, whereas the JR strategy did not, possibly due to the OTE causing uncertainty or overfitting errors^[36,37]^. Additionally, GPT-3.5 and GPT-4 performed better in English than in Chinese, suggesting that LLMs excel in language environments aligned with their training corpus^[38,39]^, highlighting the need for localized optimization in specialized fields.

### The operational mechanisms and current constraints of LLMs

LLMs based on the Transformer architecture use deep learning and self-attention mechanisms. They not only generate natural language text but also deeply understand its meaning, handling various language tasks^[40]^. The core of the Transformer is its multi-layer self-attention, enabling the model to focus on words at different positions to grasp the overall sentence meaning. Through multi-stage training, including pre-training, supervised fine-tuning, and alignment with human preferences, LLMs develop a general understanding of language^[41]^.

LLMs typically undergo two phases: pre-training to develop NLP capabilities and post-training to fine-tune models for specific domains. Pre-training, based on the Transformer framework, uses unsupervised learning from large, unlabeled text data to capture language patterns, enabling context understanding and semantic analysis^[42]^. Post-training further optimizes models through fine-tuning, improving performance for specific tasks^[43]^, especially in Few-Shot Learning scenarios, where overfitting is a concern^[44,45]^. However, clinical reasoning, such as judgment, situational awareness, and risk assessment, depends on practical experience, not just text-based learning^[46]^. In anesthesiology, particularly in crises, rapid decision-making requires advanced reasoning, causal analysis, and counterfactual thinking^[13,47,48]^. Our results show that LLMs perform worse on clinical and high-order reasoning tasks compared to non-clinical or first-order ones. Error analysis reveals that common logical errors, like misjudging causal relationships and broken reasoning chains.

DeepSeek stands out for its low cost and performance comparable to ChatGPT, particularly in clinical scenarios, high-order reasoning, and challenging subspecialty tasks. Its effectiveness stems from several advanced techniques, including targeted medical data curation, reinforcement learning (RL), and model distillation, which enhance reasoning efficiency while mitigating overfitting^[49,50]^. Additionally, as an open-source model, DeepSeek benefits from continuous community-driven improvements and efficient parameter tuning, resulting in strong performance with reduced computational overhead^[33]^. A key factor behind DeepSeek-R1’s superior performance in complex reasoning tasks is its architecture and training strategy. The model leverages RL, a technique that enables it to continuously improve its decision-making capabilities by learning from feedback. Early-stage fine-tuning with high-quality Chain-of-Thought data during the RL process has significantly enhanced the model’s ability to generate logically coherent outputs^[51]^. Furthermore, DeepSeek-R1 employs reasoning-oriented RL, rejection sampling, and supervised fine-tuning strategies to refine its performance, particularly for tasks requiring complex, extended logical chains, such as those encountered in clinical decision-making and specialized medical tasks^[52]^. Regarding its optimization for processing Chinese medical language, DeepSeek-R1’s performance is notably enhanced by its larger proportion of Chinese pretraining data. Although the exact ratio of Chinese to English data has not been disclosed, DeepSeek-V2, the predecessor of DeepSeek-R1, contained 1.12 times more Chinese tokens than English tokens^[53]^. This indicates that the model’s design, including its data curation strategy, is specifically optimized for Chinese medical terminology, leading to better results on Chinese-language tasks compared to English-language tasks.

Nevertheless, studies have shown that LLMs may experience overthinking during reasoning, generating intermediate steps unrelated to the answer, and repeated questioning may lead to fluctuations in the reasoning process, resulting in inconsistent answers^[17,18]^. Research from Microsoft suggests that with increased dialogue rounds, context overload can cause intermediate forgetting, leading to a decline in performance^[19]^. Additionally, when LLMs lack knowledge, cognitive uncertainty during repeated questioning or probing can generate contradictory responses^[20]^. This also explains why the JR strategy used in this study actually reduced the accuracy.

### Impact of language differences on model performance

The choice of question language affects model response quality, with this impact linked to the balance of the training corpus. Previous studies have shown that GPT-4 performs better in English queries related to cardiovascular disease prevention, highlighting potential language biases in models^[54]^. Syntax complexity is a key factor influencing model performance. While multilingual models have achieved notable success in cross-linguistic tasks, they still face challenges with syntactic complexity and insufficient language-specific training when processing non-English languages^[55]^.

In medical diagnostics, linguistic diversity leads to differences in information expression, with variations in terminology and symptom descriptions across languages potentially causing biases in model understanding and judgment of the same case. This underscores the challenge posed by different languages in expressing knowledge and concepts for LLMs^[56]^. In practical applications, LLMs also face ethical challenges, including AI hallucinations, information bias, privacy and data risks, and lack of transparency and explainability^[57]^. These issues are more complex in multilingual environments, where varying patient data privacy laws and cultural norms across languages require models to follow different rules, complicating privacy protection. Furthermore, performance discrepancies across languages may contribute to educational inequities, as students from certain linguistic backgrounds may not receive accurate learning support due to the model’s limited processing ability in their language.

Enhancing LLMs’ semantic understanding and overall performance in multilingual settings is crucial. This requires expanding the training data to include more medical texts in different languages and improving the semantic understanding algorithms to better handle complex semantic relationships across languages, ensuring fairness and applicability in multilingual contexts.

### Clinical implication

LLMs may struggle with complex logical reasoning, multi-factor analysis, or integrating deep domain knowledge, limiting their ability to provide accurate solutions in such cases. While LLMs have made strides in high-order reasoning, they still fall short in simulating anesthesiologists’ decision-making in complex clinical scenarios. Error analysis revealed that over half of the mistakes were high clinical risk errors, directly impacting patient safety and treatment outcomes.

In some complex medical diagnoses, LLMs fail to integrate various patient factors, such as symptoms, medical history, and test results, leading to inaccurate diagnoses. In emergency medicine, LLMs outperform traditional models in predicting clinical deterioration and providing decision support^[58]^, but their diagnostic accuracy remains lower than expert physicians. A study on abdominal disease diagnosis showed that advanced LLMs were inadequate in diagnostic accuracy, guideline adherence, and interpreting lab results, posing significant risks to patient health^[59]^. Additionally, LLMs often miss critical information, compromising diagnostic reliability.

Although the limitations of LLMs in complex clinical decision-making, their role in medical education, particularly within standardized residency training, is drawing increasing interest. LLMs can generate high-quality mock exams and provide real-time feedback on reasoning, helping to identify knowledge gaps and cognitive biases that support personalized learning. They may also be integrated into bedside teaching to offer diagnostic suggestions based on clinical data, assisting junior physicians in developing structured clinical reasoning^[60]^. Moreover, LLMs can aid in analyzing case trajectories and simulating alternative decision pathways, which enhances planning and judgment^[61]^. Collectively, these applications position LLMs as interactive cognitive tools with pedagogical value, even before they are ready for autonomous use in high-stakes clinical settings.

### Limitations

This study has several limitations. Owing to copyright restrictions, the official CAAPE questions were not accessible; instead, the NHCC-approved 2025 CAAPE item bank was used, containing 5647 text-only questions aligned with the exam syllabus. As the item bank includes no visual content and GPT-3.5/DeepSeek-V3 do not support image inputs, image- and table-based questions were not assessed. Second, given the rapid evolution of LLMs, only four versions from two representative models were evaluated, selected for their prominence in international and Chinese contexts, respectively. Finally, owing to the large sample size, direct comparison with human performance on the same items was not feasible. However, to estimate passing potential, accuracy was converted into standardized scores reflecting LLM performance in the anesthesiologist exam context. Future research should include more LLMs and human candidates to improve generalizability and reliability.

## Conclusion

DeepSeek-R1 outperformed others in accuracy and reasoning, with GPT-4 demonstrating balanced capabilities and GPT-3.5 excelling in speed. The SR prompt strategy enhanced accuracy, while repeated questioning impaired it. Models performed better in their native training language. Despite their potential in clinical reasoning, LLMs still risk logical and informational errors, especially in high-stakes settings. Caution is essential when applying LLMs in medical education and decision support to ensure patient safety.

## Data Availability

Data are available from the corresponding author if a justification for the requirement is provided.

## Research registration unique identifying number (UIN)

Name of the registry: not applicable. Unique Identifying number or registration ID: not applicable. Hyperlink to your specific registration (must be publicly accessible and will be checked): not applicable.
